# Preclinical and Early Clinical Development of Tenofovir Alafenamide/Elvitegravir Topical Inserts for Effective On-Demand Vaginal and Rectal HIV Prevention

**DOI:** 10.3390/pharmaceutics16030348

**Published:** 2024-03-01

**Authors:** M. Melissa Peet, Vivek Agrahari, Meredith R. Clark, Gustavo F. Doncel

**Affiliations:** CONRAD, Eastern Virginia Medical School, Norfolk, VA 23507, USA; mpeet@conrad.org (M.M.P.); vagrahari@conrad.org (V.A.); mclark@conrad.org (M.R.C.)

**Keywords:** HIV pre-exposure and post-exposure prophylaxis, topical PrEP and PEP, antiretrovirals, acceptability, safety

## Abstract

HIV/AIDS remains a global public health issue, and products available for the prevention of HIV infections are limited, especially those for short-acting, on-demand, user-controlled applications. Topical inserts are products that can be applied vaginally or rectally and have been explored as drug delivery systems. To fill the gap in the HIV prevention product pipeline, CONRAD has developed a topical insert containing tenofovir alafenamide fumarate (TAF) and elvitegravir (EVG), two potent and synergistic antiretrovirals, as a simple, low-cost, and discreet option that can be self-administered vaginally and/or rectally, before and after coitus. In this review, we have described the development path of the TAF/EVG insert up to its current point in clinical testing, highlighting findings from acceptability, preclinical safety, pharmacokinetics, and efficacy evaluations and early clinical studies. In summary, the TAF/EVG inserts are stable, easy to manufacture, low-cost, acceptable, and show highly promising preclinical and clinical results for on-demand topical pre- or post-exposure HIV prevention.

## 1. Introduction

The goal of the HIV Prevention 2025 Roadmap is to end AIDS as a public health threat by 2030 and reduce new HIV infections to fewer than 370,000 by 2025 [1]. According to UNAIDs (2023), 1.3 million (between 1 and 1.7 million) new infections were reported in 2022 (https://thepath.unaids.org/, accessed on 7 December 2023). With the need to reduce the yearly incidence by over 1 million, in addition to continued provision of antiretrovirals (ARVs) for treatment, expanded development of HIV prevention products (pre-exposure prophylaxis; PrEP) that are not only safe and effective but are also affordable, desirable, and meet the needs of end-users is critically needed. Barrier methods (mostly male condoms) are widely available due to their affordability [2] but do not fit every user’s preferences. Oral PrEP in the form of a daily tablet containing tenofovir disoproxil fumarate (TDF) and emtricitabine (FTC) is highly effective but, particularly in women, only with high adherence [3]. This regimen is burdensome and carries with it a stigma as pills are associated with the treatment of infected individuals [4]. With more options available for HIV prevention, the higher the potential for uptake. A 2-month long-acting injectable containing the integrase inhibitor cabotegravir has proved to be safe and highly effective and was recently approved for HIV prevention [5,6,7]. This eliminates the requirement of taking oral PrEP pills every day but requires painful 3 mL intramuscular (IM) injections every 60 days. As shown time and again, people want choices and there is no one-size-fits-all product that meets the needs of everyone at all stages in their life. Some individuals want user-controlled daily pills, some long-acting injectables, while others, especially those who engage in less frequent or clustered sex prefer short-acting on-demand products [8,9,10].

Currently, the only on-demand option proven to be effective in the MSM (men who have sex with men) population is TDF/FTC using a challenging 2-1-1 schedule (two pills 2–24 h before sex, one pill 24 h after the first dose, and another pill 24 h after the second dose). This regimen is not approved by the U.S. Food and Drug Administration (FDA). Nothing comparable exists for women or people who have vaginal sex. Topically applied mucosal HIV prevention products have a high potential for safety, ease of use, low cost, and privacy and bear a broad appeal among end-users [11]. The only topical product to prevent the vaginal transmission of HIV, which is recommended by The World Health Organization (WHO) and approved in some African countries, though not approved by the FDA, is the 1-month dapivirine vaginal ring [12]. This ring, however, is restricted to vaginal application only and must be worn daily, continuously, and replaced every month. Taken together, there exists a clear need for a product that can be applied topically, on-demand, and by both women and men and is forgiving (extended window of protection), safe, and highly effective. In the category of topical products, inserts can be applied vaginally or rectally and are a promising alternative to oral and parenteral formulations for HIV prevention.

This article brings together, for the first time, all of the latest preclinical efficacy testing and recently completed first-in-human (FIH) clinical studies on acceptability, safety, and pharmacokinetics (PK) evaluating CONRAD’s dual-compartment TAF/EVG topical insert for on-demand HIV prevention. Ongoing clinical studies and future product development direction based on end-user feedback are also included. For more information on these inserts as drug delivery platforms, please see below and in the previous review [13].

## 2. Insert Platform as a Topical Drug Delivery System

Therapeutic products delivered topically via the vaginal or rectal mucosa have been around for years and used for multiple indications. Vaginal inserts on the U.S. market include, but are not limited to, Vagifem^®^ (for atrophic vaginitis due to menopause), Cervidil^®^ (for the initiation or continuation of cervical ripening in patients at term prior to labor induction), Encare^®^ (contraceptive), and Mycelex-G (for vaginal yeast infections). Examples of rectal products on the market include CANASA^®^ (suppository for the treatment of mildly to moderately active ulcerative proctitis), Diastat^®^ (gel for the acute treatment of intermittent, stereotypic episodes of frequent seizure activity), and hydrocortisone-based suppositories for use in inflamed hemorrhoids and post-irradiation (factitial) proctitis. There are a number of research groups targeting the vaginal and rectal mucosa to topically deliver agents formulated in inserts, films, gels, rings, and enema for the prevention of HIV (a complete list can be found at AVAC Prevention Products in Pipeline; https://avac.org/resource/infographic/the-future-of-arv-based-prevention-and-more/, accessed on 6 November 2023). While there are no FDA or African regulatory-approved topical on-demand products for HIV prevention other than condoms, many in the form of enemas, films, and fast-dissolving inserts are showing promising preclinical and clinical findings [14,15,16,17,18,19]. The insert platform has also been explored for other drugs/indications to provide user-friendly, low-cost, on-demand product options, especially in lower- and middle-income countries (LMICs).

**Figure 1:**
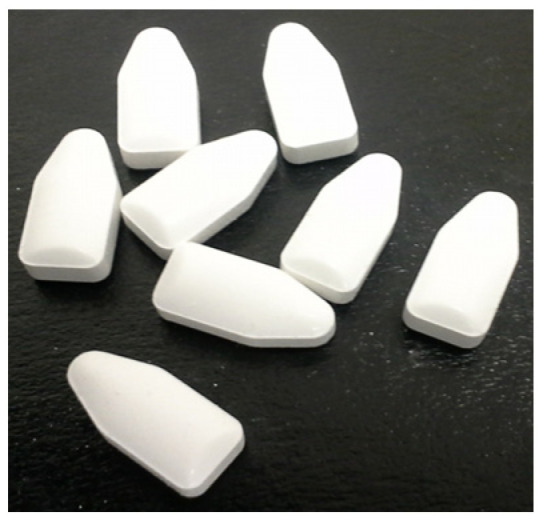
CONRAD’s current TAF/EVG insert product (1.5 [L] × 0.7 [W] × 0.6 [H] cm).

To fill the gap in the HIV prevention pipeline using topical ARV products, CONRAD is leading the development of an insert platform that can be applied using a finger both vaginally and rectally, before or after sex, is user-administered, discreet, and displays minimal side effects. This dual-compartment on-demand topical insert (Figure 1) contains TAF and EVG and is manufactured using a simple, robust, and highly scalable direct-compression process with a limited number of processing steps involved to produce inserts with high mechanical strength. The inserts comprise FDA-approved excipients from different categories, including lubricants, fillers, binders, and disintegrants in appropriate ratios to support the fast disintegration and high mechanical integrity of the final product (manuscript in preparation). The specific excipients include magnesium stearate, lactose monohydrate, poloxamers, povidone, mannitol, and polyethylene glycol [20]. When formulating inserts, multiple physicochemical characteristics are taken into consideration, such as hardness and friability, to provide a balance of fast disintegration (for immediate drug release) and appropriate mechanical integrity to withstand physical stress during the manufacturing, shipment, and usage.

## 3. Development of On-Demand Topical TAF/EVG Inserts for HIV Prevention

**Figure 2:**
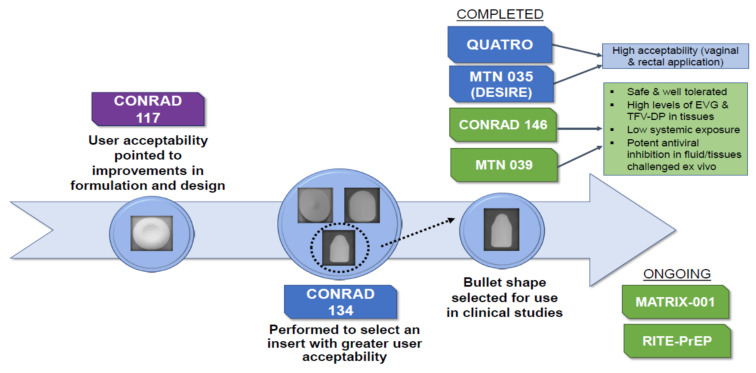
Sequence of clinical studies shaping the form and properties of the current TAF/EVG inserts, including recently completed and ongoing clinical studies.

Earlier R&D efforts of topical inserts developed by CONRAD contained tenofovir (TFV, 40 mg) and FTC (40 mg), conceptually pioneering combination ARVs for topical HIV prevention. This drug combination was based on the approved oral PrEP tablet containing TDF and FTC and the TFV 1% gel under development but in a dosage form for topical delivery, intended to be more portable and discreet than the vaginal gel. In addition to being tested preclinically, these inserts were evaluated clinically, in CONRAD 117 (NCT01694407), where they demonstrated good safety and PK profile [21,22]. User input however highlighted excessive discharge and the presence of particulate materials as two negative features that needed to be corrected before moving forward. Four different placebo inserts were reformulated for further end-user feedback in the CONRAD 134 study (NCT02534779), leading to the selection of a bullet-shaped prototype with a shape different than what had been used before and more intuitive for vaginal insertion [13,23]. All placebo prototypes had improved user acceptability, including easy use, over the earlier TFV/FTC inserts and showed a superior dissolution profile, with initial disintegration occurring within 10 min and complete dissolution as quickly as 30 min, without leaving any visible residue or triggering excessive discharge. The clinical evolution of the bullet-shaped insert, initiating with the circular insert used in CONRAD 117, is presented in Figure 2 below, which includes completed clinical studies with the placebo form (QUATRO and MTN 035) and the TAF/EVG bullet-shaped insert (CONRAD 146 and MTN 039) in recently completed and ongoing clinical studies and development.

While reformulating the platform, CONRAD explored the use of two more potent, synergistic, and mechanistically different ARVs, tenofovir alafenamide fumarate (TAF, equivalent to 20 mg of TAF free base) and elvitegravir (EVG, 16 mg), to expand the window of protection and dosing forgiveness. In vitro data using cell lines and cervicovaginal (CV) tissues supported activity under pre- and post-viral exposure conditions [24]. These new TAF/EVG inserts are manufactured using FDA-approved Generally Recognized as Safe (GRAS) materials and a simple direct-compression process, which enables low-cost [25,26], scalable, and readily transferable production of robust inserts, resulting in better handling and packaging feasibility of the final product. The accelerated and long-term stability data of these inserts provide a projected shelf life of >2 years if stored at or below 30 °C/65%RH, ensuring that the integrity, quality, and potency of the product is maintained for an extended duration. These inserts have undergone extensive acceptability, preclinical, and clinical testing to date supporting both vaginal and rectal use and a wide window of protection against HIV. The following sections discuss these aspects in detail.

## 4. Acceptability Assessment of Placebo and ARV-Containing Inserts

User acceptability is paramount to the uptake and adherence of HIV prevention interventions and their ultimate success. Acceptability assessments of products for HIV prevention following actual use have been limited, especially for rectal administration, with most being surveys inquiring about theoretical preferred use [27,28,29,30,31]. CONRAD’s topical insert in placebo form has had the benefit of undergoing acceptability testing following vaginal use [23,32] and rectal use [27] to guide product development and ensure suitability for its users. Early on in insert development, the CONRAD 134 study (NCT02534779) was conducted to evaluate variations in the composition, technology type (direct-compressed versus freeze-dried), shape, and size of different prototypes for their disintegration time, residue/leakage, and acceptability after a single use to select a lead insert to advance into clinical development (reviewed in Peet 2019, [13]). The placebo form of the lead insert has also been evaluated for acceptability in comparison to other topical vaginal and rectal placebo products [27,32], as well as in Phase 1 studies following vaginal and rectal use of the TAF/EVG inserts [33,34], as summarized below.

The placebo insert was evaluated for its acceptability after vaginal application along with three other vaginal placebo products (film, gel, and ring) in QUATRO, a mixed-method crossover clinical study performed from June 2016 to 2017 in 178 HIV prevention product naïve, sexually active women living in Zimbabwe and South Africa [32]. Products were first assessed for preference prior to actual use, resulting in 41% preferring the gel, 19% the film, 15% the ring, and 25% the insert. After a period of using each of the products, in a randomized way, at least once weekly for one month prior to or independent of sex, the film, ring, and insert were preferred by 29%, 28%, and 26%, respectively, and the initially preferred gel by only 16% after use. At the end of the study, product choice varied by country with 45% in Zimbabwe selecting the film as most preferred and 35% in South Africa selecting the insert. A qualitative component was included in the QUATRO study to gain additional perspectives from women participants (*n* = 40), their male partners (*n* = 17), and key informants (KIs; *n* = 24) [35]. Focus groups discussions were conducted with the women participants and their male partners, and in-depth interviews (IDIs) were conducted with KIs. Key findings revealed that the insert was one of the two (film noted as the other) most preferred products for women in South Africa, men in Zimbabwe, and KIs in both countries. Noteworthy positive attributes related to the insert included female-initiated application, small and easy to use, tightened vagina/enhanced sexual pleasure, familiarity (similar looking to pills), discreet, easily dissolves, shape shows how to insert, and allowance for barrier-free sex. Negative attributes were wettened vagina/reduced sexual pleasure, short-term use, stigma (package and pill-like form similar to antiretroviral therapy), method of insertion may cause infection, and too small to insert properly. It should be noted that while short-term use was listed as a negative attribute, overall, the on-demand products (insert, film, and gel) were most liked.

These inserts were also included as one of the three placebo products (insert, suppository, enema) evaluated for rectal use prior to receptive anal intercourse (RAI) for the prevention of HIV in MTN-035 (NCT03671239), a randomized crossover trial for assessment of safety, acceptability, and adherence [36]. Participants (*n* = 217) included HIV-uninfected transgender men, transgender women, and cisgender MSM from five different countries (Malawi, Peru, South Africa, Thailand, and the U.S.). Inserts were reported as safe with high acceptability and adherence in general and when compared to the other product modalities. High acceptability was reported by 72% of participants for inserts, 66% for suppositories, and 73% for the enema. The percentage of participants fully adhering to the protocol (used at least once per week) was 75%, 74%, and 83% for the inserts, suppositories, and enema, respectively. Full adherence per RAI act was comparable across products: 58.9% for the insert, 58.0% for the suppository, and 58.8% for the enema.

Acceptability of the clinical form of the TAF/EVG insert for vaginal use was assessed in 16 healthy, HIV-uninfected women in the U.S. at low risk of HIV acquisition in CONRAD 146 (NCT03762772), a Phase 1 open-label randomized, parallel-group study [33]. Following a single intravaginal administration of the insert, participants completed a quantitative survey to assess acceptability across multiple attributes of the insert at 4 or 24 h post insertion. Size, ease of insertion, and color were considered very or somewhat acceptable in 100% of participants. Comfort after insertion, dissolvability, and discreetness were also highly acceptable for 15 of 16 participants (93.8%). Acceptability of residue and leakage ranked lower (81.3% and 81.5%, respectively) but remained acceptable in over half of the participants. A few participants described leakage as a small amount of whitish, odorless discharge. A total of 7 of 16 participants (43.8%) noted that using an applicator would make it easy to administer.

The clinical formulation of the TAF/EVG insert for rectal use was also assessed for acceptability in MTN 039 (NCT04047420), a Phase 1 open-label, multi-site, single-arm, two-period study in 23 healthy, HIV-uninfected male and female participants at two sites in the U.S. Although this study was focused on safety, PK, and pharmacodynamics (PD), acceptability was evaluated using computer-administered self-interviews (CASIs) prior to and after rectal administration of one or two inserts and a concluding IDI after final use of the product. Nearly all participants found the insert easy to use and comfortable. This clinical trial was recently completed, and a manuscript is pending review [34].

## 5. Preclinical Testing of TAF/EVG Inserts Shows Safety and High Efficacy When Applied before and after Virus Challenge

TAF/EVG inserts have undergone rigorous preclinical testing to verify local mucosal safety, characterize multi-compartment PK, and establish a predicted window of efficacy for advancement to clinical testing. Using the rabbit, a sensitive and preferred model to assess mucosal irritation and local tolerability and safety [37,38], insert safety was confirmed after several weeks of repeated daily intravaginal and intrarectal doses of pre-dissolved inserts using immunohistochemical and microscopic evaluations of mucosal tissues [13,21]. Ex vivo testing was performed to aid in defining the window of protection prior to testing in a primate model. CV explant tissue treated with TAF and EVG pre-exposure, during, or post-exposure to HIV-1BaL reduced p24 antigen production at all timepoints tested (up to 24 h pre-exposure and 48 h post-exposure) [24].

**Figure 3:**
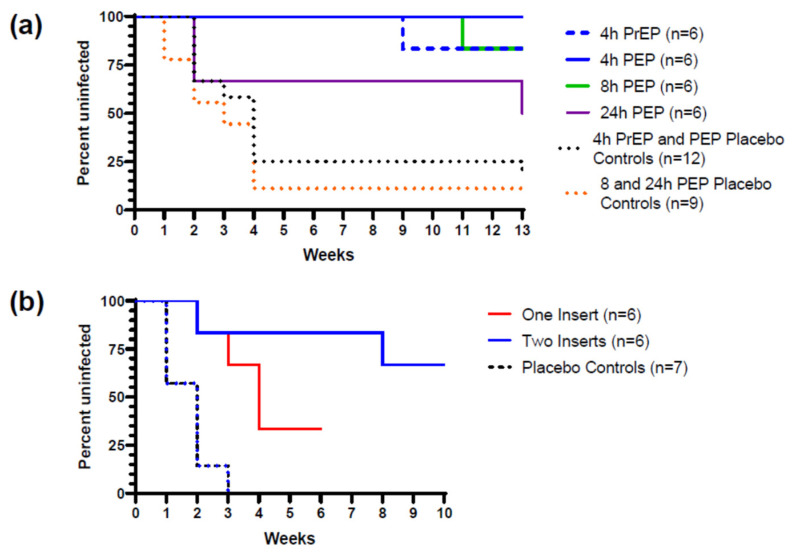
(**a**) Percent of uninfected macaques administered TAF/EVG inserts vaginally at 4 h prior to and 4, 8, and 24 h post-SHIV exposure (reproduced and modified with permission from [18,40]). (**b**) Rectal efficacy (percent uninfected) of one TAF/EVG insert vs. two TAF/EVG inserts administered 4 h before SHIV exposure in macaques (reproduced and modified with permission from [19]).

Using the repeated low-dose simian HIV hybrid (SHIV) challenge model along with multi-compartment PK assessments, TAF/EVG inserts have been evaluated to predict the clinical window of protection when used under PrEP and/or post-exposure prophylaxis (PEP) conditions and correlate drug levels with efficacy. This model has been used to predict clinical efficacy of FDA-approved ARVs for the prevention of HIV [39]. Through a collaboration with the Centers for Disease Control and Prevention (CDC), the prophylactic potential of inserts was tested against vaginal or rectal transmission of SHIV. TAF/EVG inserts at the clinical dose (20/16 mg) and administered vaginally to NHPs 4 h pre- or 4 h post-repeated weekly vaginal SHIV exposures provided protection in 5/6 animals pre-exposure and 6/6 animals post-exposure resulting in an efficacy of 91% and 100% for PrEP and PEP, respectively [18] (Figure 3a). PK evaluations confirmed rapid penetration of both drugs in vaginal tissue with EVG tissue levels above target at 2, 4, and 24 h and rising TFV-DP reaching target levels at 24 h and remaining high for at least 72 h. The efficacy of TAF/EVG inserts at 8 and 24 h post-SHIV exposure was also tested using the same challenge model, registering 94% and 72% efficacy at 8 h and 24 h post-viral exposure, respectively [40] (Figure 3a). Time to infection was delayed for animals infected with the TAF/EVG insert compared to placebo controls. Two of the animals which became infected tested positive only after 11 challenges. Using a similar methodology, the inserts were also tested for their efficacy against rectal SHIV transmission when administered 4 h pre-exposure followed by weekly repeated challenges [19]. In order to achieve better coverage of the recto-sigmoid compartment and greater than partial protection, we found that two inserts were required. Similar to what has been observed with topical TFV [41], higher drug concentrations were required in rectal tissue compared to vaginal tissue to provide protection with the inserts. When compared to single-insert application, the two-insert dose increased drug levels in rectal tissues by 10-fold, with an efficacy of 93.1% (Figure 3b).

## 6. Clinical Testing of TAF/EVG Inserts Demonstrates Safety, Pharmacokinetics, and Pharmacodynamics Profiles Compatible with Protection against HIV

**Figure 4:**
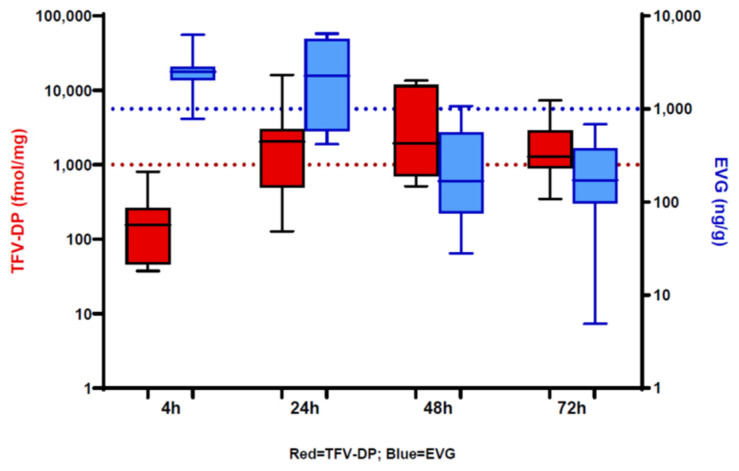
Concentrations of TFV-DP (red) and EVG (blue) in vaginal tissue at multiple timepoints post dose (reproduced and modified with permission from [33] under the terms and conditions of the Creative Commons Attribution (CC BY) license). Red dotted line represents TFV-DP target levels of 1000 fmol/mg, and blue dotted line represents target EVG levels of 1000 ng/g.

TAF/EVG inserts have been evaluated in two Phase 1 studies following vaginal (CONRAD 146) or rectal (MTN-039) administration. The clinical manufacturing of inserts under cGMP (Current Good Manufacturing Practice) conditions was conducted at Patheon, Inc., (Whitby, ON, Canada). CONRAD 146’s primary objective was to assess safety and tolerability, multi-compartment PK, and PD out to 7 days following a single vaginal application of the TAF/EVG insert in 16 healthy women in the U.S. [33]. Safety was confirmed using treatment-emergent adverse events (TEAEs); no TEAEs were observed after a single dose of the insert. Vaginal administration was well tolerated and resulted in low plasma levels and no systemic side effects. PK and PD evaluated in vaginal fluid and tissue correlated well and supported an extended window of protection, revealing rapid delivery of both drugs at the earliest timepoint of collection, 4 h, and high levels out to 72 h post dose. The combination of TAF and EVG works well as EVG has a fast onset with rapid uptake into tissue, while TAF lasts longer, requiring conversion to TFV and TFV-DP. EVG vaginal tissue concentrations were above target protection levels (1000 ng/g; more than 20× EVG PA-IC95 [45 ng/mL] [42]) at 4 h and maintained out to 24 h, similar to what was seen in NHPs. Also, similarly to NHPs, TFV-DP levels were detected at 4 h post dose and were above the target benchmark (1000 fmol/mg) [43] at the 24, 48, and 72 h collection timepoints (Figure 4).

**Figure 5:**
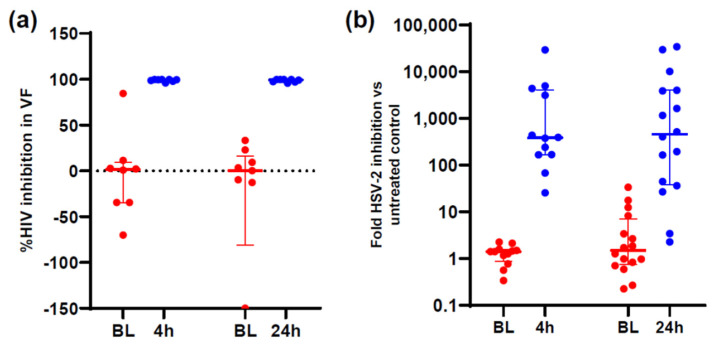
Ex vivo PD modeling of anti-viral effects in vaginal fluid. (**a**) Vaginal fluid HIV-1 inhibition. (**b**) Vaginal fluid HSV-2 inhibition. Red = baseline (BL) and blue = 4 or 24 h (reproduced and modified with permission from [33] under the terms and conditions of the Creative Commons Attribution (CC BY) license).

Cell-based and CV tissue assays revealed that the combination of TAF and EVG provided extended pre- and post-exposure inhibition of HIV replication. TAF demonstrated inhibitory activity against both HIV-1, its primary indication, and HSV-2, an additional exploratory indication, at 4 and 24 h post dose in vaginal fluid (Figure 5) [33]. TFV has been shown to inhibit HSV-2 infection in vitro, in animal models, and in clinical studies [44].

MTN-039 had a similar objective of assessing the safety and tolerability, multi-compartment PK out to 72 h post application, and PD of inserts following rectal administration in 23 participants at two U.S. sites [34]. Assessments were performed after rectal administration of one versus two inserts. Preliminary findings from this study have been reported [34], confirming that the rectal administration of TAF/EVG inserts is safe and well tolerated. Adverse effects were limited to mild anal erythema following one insert in a single participant. Rectal application also resulted in low plasma levels and no systemic side effects. EVG levels in rectal tissue were high at 2 h post application (exceeding 1 ng/mg) and showed a decline by the 24 h timepoint. TFV-DP levels were maintained markedly high, especially with two inserts out to 72 h post dose. Using the ex vivo rectal challenge model, HIV-1 p24 levels were significantly lower than the baseline with both one and two inserts out to 72 h. Further details are pending the publication of the manuscript.

## 7. Summary and Future Directions for TAF/EVG Inserts

**Figure 6:**
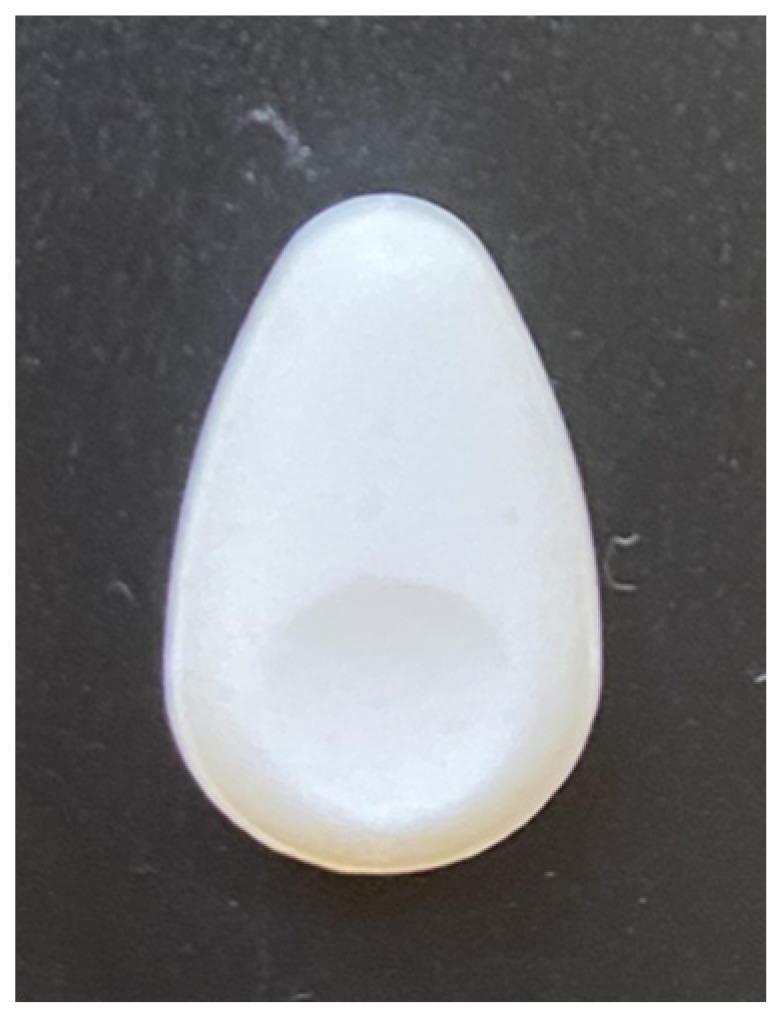
Almond-shaped insert prototype with dimple indent (1.5 [L] × 1 [W] × 0.4 [H] cm).

The TAF/EVG inserts described in this review were rationally designed and developed by CONRAD following in vitro, animal model, acceptability/end-user input, and clinical testing. At present, these inserts are the most clinically advanced topical HIV prevention product for women and/or men with potential for flexible pre- or post-exposure use. Overall, the inserts have proven to be safe and efficacious preclinically, further displaying clinical PK/PD profiles compatible with HIV protection. They have also proven to be stable, easy to manufacture, low-cost, and potentially transferable to large-scale cGMP production in LMICs. It is worth noting that the concept of combining two synergistic ARVs with different physicochemical properties and PK profiles supports a highly effective, lasting, and forgiving (i.e., with a wider window of protection) dosing regimen, all properties that have been verified in both animal and clinical studies and represent unique characteristics of this novel method of on-demand HIV prevention for all genders. The development of TAF/EVG inserts has been and continues to be an iterative process. With a strong focus on end-user preferences, other design (shape/size) options are being explored that may be more intuitive and easier for application, along with different packaging options to maintain product discreetness and portability for on-demand use. End-user feedback is ongoing to determine whether an evolution of the bullet-shaped insert design, under continued clinical development for the TAF/EVG insert, to an almond shape with a dimple indent for the placement of a finger and ease of administration, for potential future insert products in preclinical development, is preferred (Figure 6).

**Figure 7:**
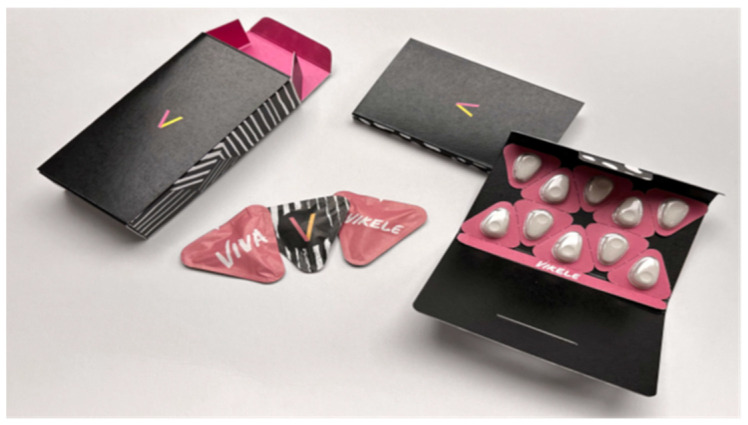
Prototype packaging designs, informed by human-centered design process, intended for discreet, portable, and on-demand use.

Exploration of individual insert packaging and packaging for multiple inserts in one is also ongoing with the goal of producing a portable, functional, and discreet product package (Figure 7).

TAF/EVG inserts will be tested vaginally for the first time in Africa in a clinical study initiating at the end of 2023/beginning of 2024 (MATRIX-001, NCT06087913). This study will enroll approximately 60 healthy, non-pregnant, HIV-uninfected women at three clinical sites in South Africa, Kenya, and the U.S. MATRIX-001 will examine the safety, PK, modeled PD, disintegration, and acceptability of inserts. Participants will be randomized (1:1) to apply either a placebo or TAF/EVG insert vaginally once daily for 3 consecutive days and then every other day (QOD) for 14 days. Another clinical study planned, but assessing rectal application, is a placebo-controlled, randomized Phase 1 clinical trial in the U.S. (RITE PrEP) to evaluate the safety and PK of two TAF/EVG inserts administered rectally for 3 consecutive days and after seven doses on alternating days in up to 24 healthy, non-pregnant, HIV-uninfected participants.

Taken together, the current findings support the continued development and potential use of TAF/EVG inserts as a desired, gap-filling, and affordable option for on-demand/event-driven, discreet, flexible, and topical PrEP and PEP regimens against vaginal and/or rectal HIV transmission.

## 8. Patents

The authors are named in a patent application on “Pharmaceutical compositions and methods of making on demand solid dosage formulations” (PCT No.: PCT/US19/57645; Pub No. US 2021/0379089 A1), an invention developed under a U.S. Agency for International Development (USAID)-funded cooperative agreement.
